# Performance characterization and application of composite adsorbent LiCl@ACFF for moisture harvesting

**DOI:** 10.1038/s41598-021-93784-7

**Published:** 2021-07-13

**Authors:** X. Y. Liu, W. W. Wang, S. T. Xie, Q. W. Pan

**Affiliations:** 1 National Defense Engineering Institute, Academy of Military Science of PLA, Beijing, 100036 China; 2 Institute of Refrigeration and Cryogenic, Shanghai Jiao Tong University, Shanghai, 200240 China; 3 Engineering Research Center of Solar Power and Refrigeration, MOE, Shanghai, China

**Keywords:** Porous materials, Hydrology

## Abstract

Freshwater scarcity is a global threat to modern era of human society. Sorption-based atmospheric water harvesting (AWH) is prospective to provide fresh water for remote water-stressed areas lacking in water and electricity. Adsorbent material plays a vital role in such AWH systems. Here, we report a solid adsorbent synthesized by impregnating hygroscopic salt lithium chloride (LiCl) into solidified activated carbon fiber felt (ACFF modified by silica sol). Composite samples immersed with different mass concentrations of silica sol are prepared and characterized for dynamic water uptake, equilibrium water uptake, textural and thermal properties. AS5Li30 (ACFF + 5 wt% silica gel + 30 wt% LiCl) exhibits an efficient water uptake of 2.1 g/g at 25 °C and 70% relative humidity (RH). The material further demonstrates a heat storage capacity of 5456 kJ/kg. Its low regeneration temperature (< 80 °C) and good cycle stability make it feasible to be used in practical water production applications, driven by solar energy and other low-grade energy. Estimation results show that water harvesting unit can produce 1.41 g_H2O_/g_AS5Li30_ under 25 °C and 75% RH.

## Introduction

Freshwater is fundamental to human society and over fifty percentage of the world’s population is experiencing water shortages to varying extents^1^. Atmospheric water harvesting (AWH), which directly captures water from the ubiquitous atmosphere, is regarded as an alternative to relieve water stress. Recently, the adsorbent-assisted AWH has been a hot research topic and has attracted significant attention of many scientists^2–6^. The adsorbent captures water vapor at higher relative humidity (RH) and then desorbs at higher temperature, condensing the released water vapor to liquid water. Proper and efficient solid adsorbent is vital to the working performance of such AWH systems. An ideal solid sorbent should feature high water uptake, fast adsorption and desorption dynamics, low regeneration temperature and good cycling stability.

Traditional solid physical porous adsorbents, like silica gels, zeolites, are usually limited by poor water uptake or high regeneration temperature. New high-performance porous adsorbent metal–organic frameworks (MOFs)^7,8^ have been very costly. Chemical adsorbents, like hygroscopic salts, indeed have higher water harvesting performance and reasonable regeneration temperature, but they usually suffer from the difficulty in desorption process caused by deliquescence and disadvantages of agglomeration, corrosion and decreasing cycling stability^9–12^.

A method for solving abovementioned problems is to impregnate hygroscopic salts into the pore space of a porous matrix^13^, called selective composite adsorbent salt@matrix. The porous matrix, usually featuring high specific surface area, is able to contain some amount of liquid solution, solving the problem of salt leakage and agglomeration. And the salt serves as the main moisture capturing role. Most widely used porous matrix includes silica gel^14–17^, ordered mesoporous silicate^18,19^, expanded natural graphite^20,21^ and activated carbon^22,23^. Composite adsorbents based on such matrix can suffer from either low water uptake, salt leakage, rupture or not being suitable for large-scale application. Recently, new porous matrix inspired the investigation of high-performance composite adsorbents, like hydrogels^24^ and aerogels which usually provide large pore volumes to contain more hygroscopic salts. However, hydrogels tend to have drawbacks of low specific surface area, and swelling after adsorbing moisture^5^. Alternatively, ACFF is intensively used in numerous applications^23–28^ like water treatment, gas separation, with advantages of extremely high surface area^29^, easy-to-be-shaped mechanical properties^25^, better adsorption characteristics, fast adsorption kinetics^30^ and uniform micropore distribution to provide strong capillary strength for water adsorption.

As for the hygroscopic salt for AWH application, calcium chloride (CaCl_2_), lithium bromide (LiBr) and lithium chloride (LiCl)^31,32^ are commonly employed in the fabrication of composite adsorbents. For example, Wang et al. studied and compared the water adsorption performance of CaCl_2_@ACF and LiCl@ACF, and the ACF-Ca30 showed a water uptake of 1.7 g/g^33^ and 0.81 g/g for ACF-Li30^34^(25 °C and 70% RH). Xu et al. reported a composite sorbent by confining LiCl in a MOF matrix LiCl@Mil-101(Cr), which shows a water sorption capacity of 0.77 g/g at 30 °C & 30% RH^11^. Xu et al., also tested and compared five typical hygroscopic salts, and reported that LiCl has higher water harvesting performance under the same adsorption condition^11^.

Herein, ACFF was chosen as the porous matrix and LiCl was screened as the active hygroscopic salt to be impregnated in the porous matrix ACFF. In addition, the silica sol was used as additional supporting material for providing stable structure as heat and mass transfer channel. By immersing pure ACFF with silica sol and after drying, the solidified matrix AS (ACFF-Silica sol) can be obtained. The objectives of this work are to fabricate some composite adsorbents and determine the optimal silica sol concentration. Firstly, we synthesized several composite adsorbent samples by impregnating ACFF with LiCl solution, with different mass concentration of silica sol. Then the samples were initially tested in a chamber with constant humidity and temperature for dynamic adsorption performance to select the optimal silica sol concentration. After that, the selected adsorbent sample was further characterized by equilibrium water uptake performance by ASAP2020 Plus, thermal properties including adsorption/desorption heat by simultaneous thermal analyzer (STA), cycle water uptake performance by STA and textural properties including BET surface area, pore volume, pore size distribution, by ASAP2020 Plus. In addition, the actual water harvesting capacity based on the selected composite adsorbent was predicted. Finally, an estimation was carried out to assess the water harvesting performance of the composite adsorbent in actual application.

## Materials and methods

### Materials fabrication

The composite adsorbents used in this work were synthesized by porous matrix ACFF with a thickness of 3 mm, silica sol (SS) and lithium chloride (LiCl). Firstly, the ACFF was dried in an oven at 100 °C for 4–6 h to fully discharge the possible remaining moisture and impurities. After cooling down to room temperature, the ACFF was immersed into the silica sol with varied mass concentrations at an ambient temperature for 1 h, sufficient for the silica sol to well distribute inside the gaps and pores of the ACFF. Then, the immersed samples were dried at 120 °C for 6–8 h until no mass changed. Thirdly, the samples were impregnated in the aqueous solution of LiCl for 8 h. Finally, dehydrate the samples again at 120 °C for 6–8 h until the mass stopped changing. In this work, silica sol with different mass concentrations, namely 0%, 5%, 10%, 15%, 20% and 30%, were used to investigate its performance in supporting the ACFF matrix and enhancing the water harvesting performance. With its help, the composite adsorbents can retain its shape after capturing moisture. LiCl was chosen as the hygroscopic salt and the mass concentration is 30%, which displays the best water uptake performance^34^. The actual proportion of the matrix, silica sol and salt in the composite adsorbents were determined by weighing dry samples before and after impregnation. The results were outlined in Table 1 and the adopted electronic scale has an accuracy of 0.001 g.

**Table 1:** Parameters of different composite adsorbents.

Sample	Silica sol concentration silica sol, wt%	LiCl concentration, wt%	Proportion of silica sol, wt%	Proportion of LiCl, wt%
AS5Li30	5		10.1	73.5
AS10Li30	10		14.8	68.7
AS15Li30	15		28.9	56.5
AS20Li30	20		38.2	47.5
AS30Li30	30		58.7	33.3
AS30Li0	30	–	92.0	–

### Characterization methods

#### Water sorption performance measurement

Dynamic water adsorption performance was tested in the constant temperature and humidity chamber (Binder KBF115, Germany). An analytical balance was adopted to weigh and record the real-time weight of the materials. Its draft shield guarantees the weighing accuracy. Meanwhile, the water adsorption isotherms of the composite adsorbents were obtained by the Micromeritics ASAP2020 Plus Surface Area and Porosity Analyzer. Its detailed compositions and measuring principle can be referred to Ref.^35^. Both dynamic and isothermal water uptake test include 2 processes: i.e., samples preparation and measurement. During the samples preparation process, the adsorbent samples are firstly heated in an oven at 120 °C for 4 h to discharge the remaining moisture. Then, the measurement process begins.

#### Surface properties measurements

The micrograph of the composite adsorbent was observed by scanning electron microscopy (SEM). ASAP2020 Plus was used to measure the porous parameters, like specific surface area, pore size distribution and pore volume through standard nitrogen adsorption/desorption at 77 K^36^. The specific surface area is calculated by BET (Brunauer–Emmett–Teller) equation and the pore size distributions were measured based on the nitrogen-desorption branches of the isotherm by applying BJH (Barret-Joyner-Halenda) method and density functional theory (DFT).

#### Thermogravimetric analysis

The coupled heat flow and mass change during the adsorption and desorption processes are recorded by the simultaneous thermal analyzer (Netzsh STA 449 F3), equipped with a moisture humidity generator (MHG 32, ProUmid). The desorption temperature rises from room temperature to 80 °C with a heating rate of 2.5 K/min and is maintained for 2 h at 80 °C. After the completion of desorption, the temperature decreases from 80 °C to room temperature (~ 30 °C) and simultaneously the adsorption occurs under 30 °C & 70% RH for about 9 h. During the test, the mass and heat flow changes are recorded simultaneously. Water uptake, in the unit of g/g, is calculated based on the dry mass at the end (beginning) of the desorption (adsorption). The adsorption/desorption heat is obtained by integrating the curve of heat flow. The purge gas is 10 ml/min.

## Results and discussion

### Dynamic water uptake performance

**Figure 1:**
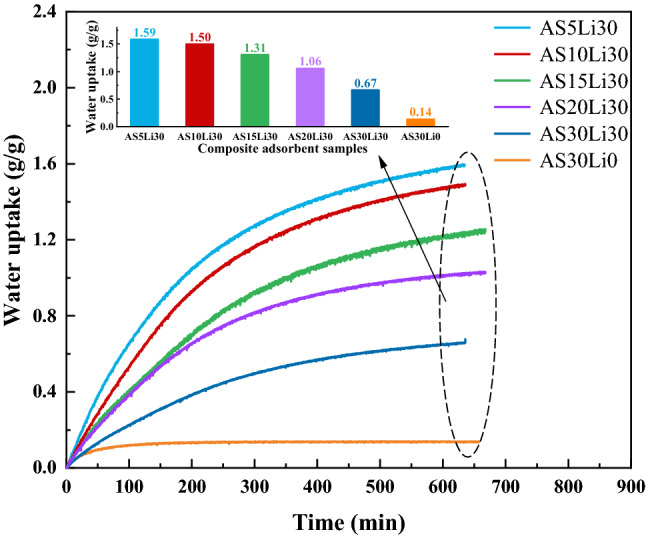
Dynamic adsorption performance of different adsorbent samples under 25 °C and 60% RH.

The dynamic water uptake performance of the composite adsorbent samples at 25 °C and 60% RH are shown in Fig. 1. The ordinate represents the water uptake performance. The initial mass is obtained from the samples dehydrated at 120 °C for 6 h. An electronic balance is adopted to weigh the samples in real time. The total adsorption time is around 12 h. In Fig. 1, the adsorption rate of the samples reaches its highest at the beginning and then gradually decreases as the adsorption progresses. Taking the AS0Li30 as an example, the water uptake at 200 min (1.05 g/g) reaches 65.9% of its saturated water uptake (1.59 g/g). This is due to the occurrence of chemisorption initially, followed by slow process of deliquescence and salt solution absorption. The adsorption curve of AS30Li0, containing no LiCl, verifies this phenomenon. It displays a fast-physical adsorption process. Unsatisfactorily, the saturated water uptake is only 0.14 g/g. So LiCl is impregnated to enhance its water harvesting performance.

It is also clear from Fig. 1 that AS5Li30 displays the best adsorption performance, meaning that the smaller silica sol content, namely higher salt content, the greater water uptake performance. For example, the saturated water uptake of AS5Li30 is 1.59 g/g, nearly 2.4 times that of AS30Li30 (0.67 g/g). The reason for impregnating with silica sol is that the ACFF porous matrix is too soft and prone to crack, while the silica particles adhere on the fibers and aggregate in the fiber gaps to support the matrix and extend the surface area for salts to adhere. However, the higher silica sol content means smaller pore volume, smaller salt concentration, as well as smaller water uptake. Thanks to the huge surface area, no salt leakage was observed for all the samples under the experimental conditions.

**Figure 2:**
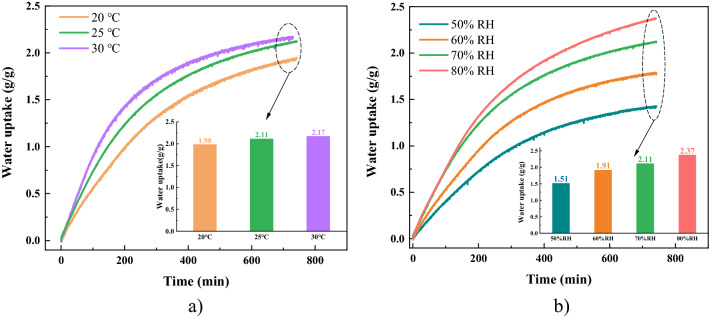
Dynamic water uptake performance of AS5Li30. (**a**) Water adsorption dynamics under 20 °C, 25 °C, 30 °C & 70% RH; (**b**) Water adsorption dynamics under 25 °C & 50% RH, 60% RH, 70% RH and 80% RH.

Therefore, AS5Li30 is selected as the composite adsorbent for water harvesting and further characterized to study the influence of operating conditions (e.g., temperature and relative humidity) on its water uptake performance, as shown in Fig. 2. It can tell from the picture that: 1) under the same RH, the higher temperature, the greater water uptake performance. But this enhancement is not very significant. 2) Under the same temperature, with the increase of RH, AS5Li30 displays greater water uptake. For example, it harvests 2.37 g/g water at 25 °C & 80% RH, which is almost 1.6 times higher than 1.51 g/g at 50% RH. This indicates that AS5Li30 is more sensitive to RH, making it extremely ideal to wide-RH applications.

Besides, we also compare the water uptake of AS5Li30 with some adsorbents mentioned in the literature, as listed in Table 2. It is clear that the proposed absorbent AS5Li30 is unmatched under the same adsorption condition.

**Table 2:** Comparison of water uptake of different adsorbents.

Adsorbent	Adsorption condition		${\text{Water uptake}}/{\text{g}}_{{{\text{water}}}} \;{\text{g}}_{{{\text{adsorbent}}}}^{{ - {\text{1}}}}$
	Temperature/°C	RH/%	
ACF-Ca30^32^	20	70	1.60
ACF-Li30^33^	25	70	0.81
LiCl-Mil-101(Cr)^10^	30	30	0.77
AS5Li30	20	70	2.01
	25	30	0.83

### Establishment of models for equilibrium water uptake performance

**Figure 3:**
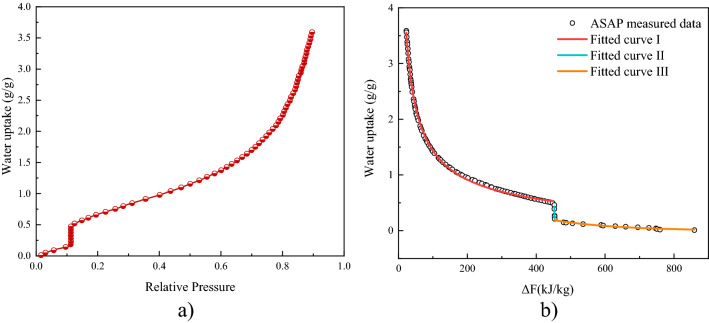
Water adsorption curves of AS0Li30 under 25 °C. (**a**) Water adsorption isotherm measured by ASAP; (**b**) characteristic ΔF -W water sorption curves. Hollow dotted data (experimental) and solid line (fitting from).

Water adsorption isotherm of AS5Li30, measured by ASAP2020 Plus at 25 °C, is illustrated in Fig. 3. The ordinate indicates the water uptake performance. The initial weight was gained from the sample dried at 120 °C for 6 h. AS5Li30 shows a type II water sorption curve with a stepwise position at small pressure (~ 0.11), which is the DRH (deliquescence relative humidity) of LiCl^20^. This indicates the formation of salt hydrate and the occurrence of deliquescence. After this RH transition region, the water uptake increases significantly with the RH, and a maximum water uptake exceeds 3.6 g/g at 25 °C & 90% RH.

Besides, for further simulation investigation, the characteristic ΔF -W water sorption curve (see Fig. 3b) was obtained by applying Polanyi adsorption potential theory, which has been widely used in analyzing sorption equilibriums of various adsorbents^37–39^. The sorption potential, also called free sorption energy, is defined as:

1 $$\Delta F = - RT\ln \frac{{P_{{{\text{sat}}}} }}{P}$$

where, *P* is the vapor pressure (Pa), ${\text{P}}_{\text{sat}}$ is saturated vapor pressure at adsorption temperature (Pa). Then, the D-A (Dubinin-Astakhov) isotherm equation, described as Eq. (4), is applied to simulate the water uptake of AS5Li30.

2 $${\text{W}} = {\text{x}}_{0} \exp ( - {\text{k}}\Delta F^{n} )$$

where, ${x}_{0}$, k, and n are the fitting parameters and the values of them can be found in Table 3. As Fig. 3 b shows, the characteristic curve can be divided into 3 segments I-III. Segments I and III are fitted by D-A equation and segment II is described by a linear fitting equation:

3 $$W = {\text{c}} - {\text{d}}\Delta F$$

**Table 3:** Fitted curve for equilibrium adsorption of AS5Li30.

Sample	Segment	$\Delta \;{\text{F}}\;{\text{(kJ/kg)}}$	Fitted equation	R^2^
	I	22.8–447.6	${\text{w}} = 4.8454 \times 10^{5} \;\exp ( - 10.1076\Delta {\text{F}}^{{0.0501}} )$	0.9988
AS5Li30	II	447.6–453.7	${\text{w}} = - 0.2256\Delta {\text{F}} + 102.6125$	0.9352
	III	453.7–859.4	${\text{w}} = 1.3879\exp ( - 0.0016\Delta {\text{F}}^{{1.1666}} )$	0.9570

In which, c and d are fitting parameters, listed in Table 3 as well. R^2^ is the square of the multiple correlation coefficient and it is used to evaluate the fitting quality. Results show that segment I fitted by D-A equation have the best agreement with the measured data, with a R^2^ of 0.9988. Yet the fitting goodness of segment II and III are not as good as I, with a R^2^ of 0.9352 and 0.9570 respectively, it is still acceptable and convincing for water uptake simulation study.

### Thermal characterizations

**Figure 4:**
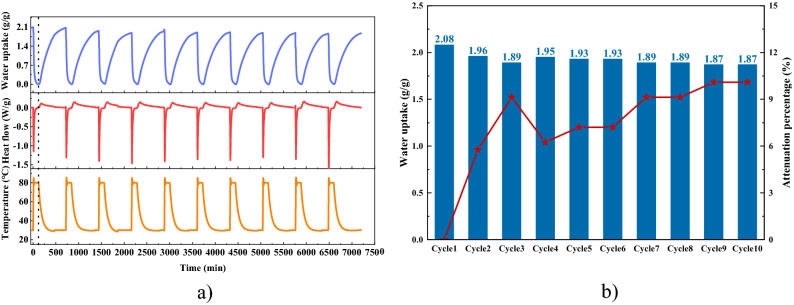
STA results of AS5Li30, adsorption at 30 °C and 70% RH, desorption at 80 °C.

Sample AS5Li30 was tested by STA for thermal characterization and cycling water harvesting stability investigation. Results are shown in Fig. 4, the water uptake, heat flow and temperature are recorded simultaneously during the test. When the desorption starts, the sample begins to lose weight until it is fully desorbed at 80 °C. There is one peak occurring in the curve of heat flow during the desorption process, which happens at 85.3 °C. This is because of the release of water from the solution and the presence of LiCl∙H_2_O, meaning that a large proportion of heat is stored in the crystallization and deliquescence process. The water uptake curve shows a maximum water harvesting capacity of 2.1 g/g under 30 °C and 70% RH. It also confirms that the adsorbent AS5Li30 can be fully regenerated at 80 °C, which can be triggered by solar energy and other renewable energy or low-temperature heat sources. By integrating the heat flow curve with time, the desorption and adsorption heat are obtained to be 5465 kJ/kg and 5054 kJ/kg, respectively.

Besides, the cycling water harvesting performance of AS5Li30 was also investigated by STA. An adsorption–desorption cycle includes a 2-h desorption process at 80 °C and then a 10-h adsorption process at 30 °C and 70% RH. Totally 10 cycles were performed, shown in Fig. 4. The water uptake of the first cycle is higher (2.08 g/g, Fig. 4b) because the test started with a fully saturated AS5Li30. Considering the fluctuation of test conditions and the measurement error, AS5Li30 displays a good cycle stability in spite of a maximum attenuation of 10%. This means a long-term service life once it is used in actual water harvesting applications.

### Textural properties

**Figure 5:**
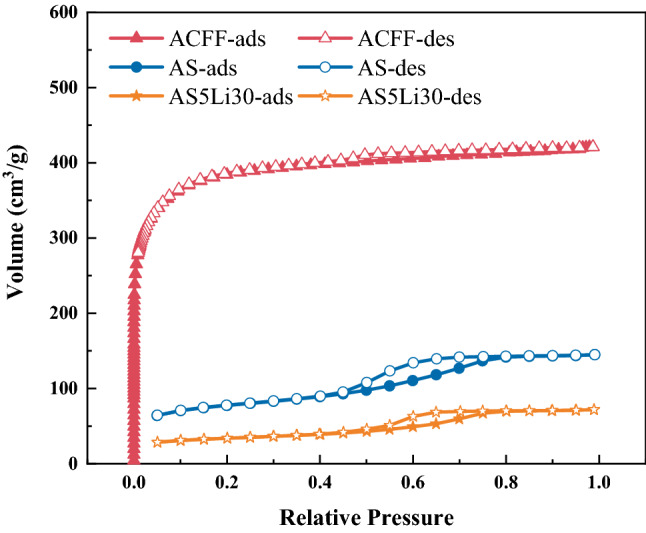
Nitrogen adsorption isotherms at 77 K.

The surface properties of the matrix and composite samples, such as isotherms by nitrogen adsorption, BET surface area, pore volume and average pore size, are displayed in Fig. 5 and Table 4. Figure 5 shows the nitrogen sorption and desorption isotherms of matrix ACFF, AS (solidified matrix composed by ACFF and silica sol) and AS5Li30. ACFF matrix shows type I isotherms with no hysteresis phenomenon, which means the dominance of micropores. However, for AS and AS5Li30, type IV isotherms with hysteresis loops of type H2 within a relative pressure range of 0.45–0.75 are observed. In addition, a sharp increase in nitrogen sorption performance occurs at the beginning of the hysteresis loop, indicating the capillary condensation and the contribution of mesopores. Moreover, AS and AS5Li30 show much less nitrogen sorption than ACFF. This is contributed to the impregnation of silica sol and salt particles, which blocks the pores of ACFF.

**Table 4:** Specific surface area and pore volume.

Sample	BET surface area, m^2^/g	Total pore volume, cm^3^/g	Average pore diameter, nm
ACFF	1376.782	0.651	1.787
AS	286.998	0.224	3.120
AS5Li30	124.222	0.111	3.578

The surface area and pore parameters are outlined in Table 4. The calculated BET surface area of ACFF is 1376.782 m^2^/g, nearly 11 times of that of AS5Li30. Whereas, BET surface area is not the key factor to water uptake but is responsible for heat transfer in composite adsorbent. Clearly, both BET surface area and pore volumes of composite adsorbent AS5Li30 and AS are much less that those of pure matrix ACFF. This is due to that the adding of silica sol and hygroscopic salt blocks the pores. Besides, due to the complex characteristics of salt assembling when it depositing on the silica gel particles^38,40^, the average pore size can either increase or decrease. Here, the average pore sizes of AS and AS5Li30 are found to be larger than that of the pure matrix ACFF, partly due to the blocking of narrow pores.

**Figure 6:**
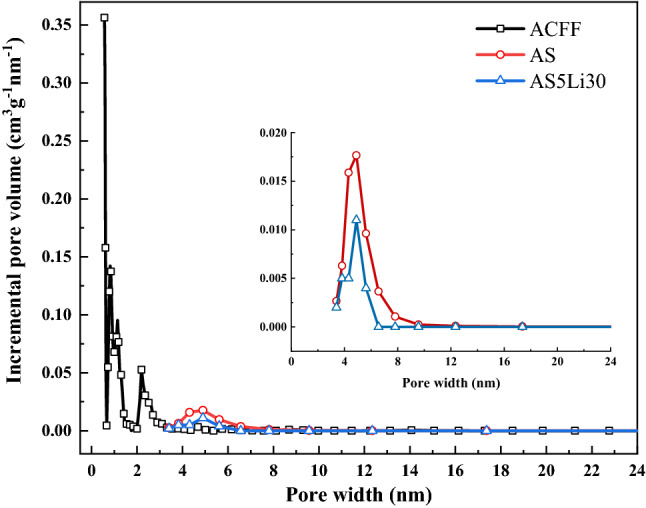
Pore size distributions. ACFF’s pore size distribution was obtained by DFT method while AS and AS5Li30 were applied with BJH method.

In addition, to better understand the pore structures of composite adsorbent AS5Li30, Fig. 6 displays the pore size distributions of ACFF, AS and AS5Li30. A multi-peak and a sharp-decrease phenomenon are observed in the pore size distribution curve of ACFF matrix, including the sharp decrease at the beginning of the curve in the microporous region within 0.5–0.7 nm, a peak at 0.85 nm and a peak stretching toward mesoporous region at 2.1 nm. This indicates that the ACFF matrix contains mainly micro pores, which essentially coincides with the trend of nitrogen sorption istorms. Besides, an obvious peak stretching toward the mescroporos region can be viewed in AS and AS5Li30 at about 4.9 nm. The peak vaule of AS5Li30 at 4.9 nm is less than that of AS, due to the impregnation of salt particles minishing the pore size of ACFF.

**Figure 7:**
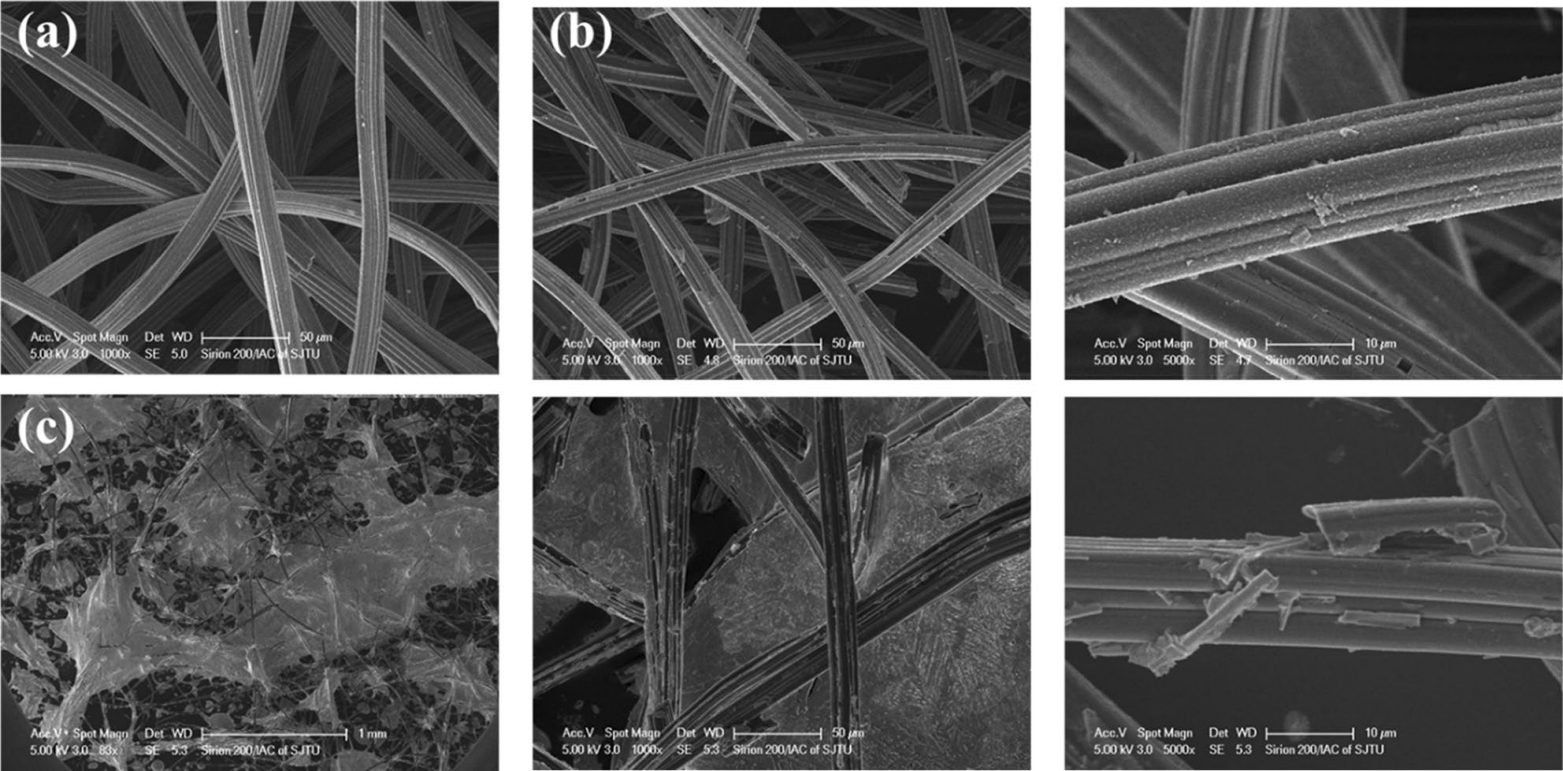
SEM micrographs of the composite adsorbent. (**a**) SEM image of ACFF matrix. (**b**) SEM images of solidified matrix AS and its enlarged SEM image. (**c**) SEM images of composite adsorbent AS5Li30 and its enlarged images with high magnification.

Field-emission SEM is used to display the microscopic morphology of the composite adsorbent, as displayed in Fig. 7. In Fig. 7a, the ACFF fibers are soft and flexible before consolidated by the silica sol. As for the AS matrix as shown in Fig. 7b, the silica grains have adhered to the fiber surface firmly to support the soft fibers. Figure 7c shows the macrograph of composite adsorbent AS5Li30. Some parts of the fibers and gaps in the figures are black, meaning that salts cannot completely cover the fiber or fill in the gaps. This is partly caused by the low mass concentration of silica sol, which provides small surface area for the salt to adhere. It can also tell from the last picture in Fig. 7c that the obtained composite adsorbent AS5Li30 is harder and more fragile than the pure ACFF.

### Water harvesting performance assessment

For real AWH application, the composite adsorbents made by AS5Li30 can be processed into flat plates and corrugated plates. Then put them layer by layer vertically and in a staggered arrangement in a case to form honeycomb-like air channel. The case can adopt acrylic sheets to prevent salt corrosion. To assess the feasibility of composite adsorbent AS5Li30 in water harvesting application, here we carry out a predication analysis. Adsorption/desorption conditions are selected form literatures^41^ to compare and estimate the water harvesting performances. The estimated water harvesting performance is listed in Table 5. For the estimation, adsorption potential ${\Delta F}$ and water uptake were calculated based on Eq. (1) and Eq. (2). By applying AS5Li30 in the water harvester in Ref.^41^, a significant increasement in water harvesting capacity can be obtained. For example, the water uptake is expected to increase from 0.55 to 1.41 g/g under condition1. Moreover, the composite adsorbent AS5Li30 displays a wide environment adaptation to be feasible even under low humidity conditions.

**Table 5:** Water harvesting performance estimation.

	Condition1		Condition2		Condition3	
	Lab^41^	Estimation	Lab^41^	Estimation	Lab^41^	Estimation
T_ads_ (°C)	25		25		15	
RH_des_	0.75		0.39		0.37	
${\Delta}(\text{F(kJ/kg})$	–	59.79	–	195.71	–	206.66
W_ads_ (g/g)	0.91	1.98	0.43	0.93	0.28	0.89
T_des_ (°C)	68		72		74	
T_cond_ (°C)	29		32.2		33	
$\Delta \text{F}(\text{kJ/kg})$	–	408.28	–	407.61	–	414.50
W_des_ (g/g)	–	0.57	–	0.57	–	0.56
W* (g/g)	0.55	1.41	0.30	0.36	0.20	0.33

*W was obtained by W_ads_ − W_des_.

## Conclusion

Composite adsorbents LiCl@ACFF were synthesized for atmospheric water harvesting. Samples immersed with different mass concentrations of silica sol are developed and tested. Characterizations including dynamic adsorption performance, equilibrium water uptake performance, thermal properties, textural properties and cycle water harvesting stability are experimentally measured and studied. The conclusions can be summarized as follows:

1. AS5Li30 is the optimal composite adsorbent for AWH, featuring the best adsorption dynamics under the same conditions, and its saturated water uptake (1.59 g/g) is 2.4 times higher than that of AS30Li30.
2. Characteristic ΔF -W water sorption curve is obtained based on the Polanyi potential theory under 25 °C, and the fitting curve agrees well with the experimental data. The characteristic curves can be used to estimate the water uptake performance of AS5Li30 for different operating conditions.
3. Experimental results by ASAP2020 Plus, such as nitrogen adsorption/desorption isotherms, BET surface area, pore volume and pore size distribution, indicate that the textural properties of composite adsorbent AS5Li30 and solidified matrix AS were different with those of pure matrix ACFF. The decrease of BET surface and pore volume of AS and AS5Li30 is due to the impregnation of hygroscopic salt particles and silica sol particles.
4. Results of STA show that AS5Li30 can be sufficiently regenerated at 80 °C, making the solar energy or other renewable energy resources applicable. The cycling water harvesting test shows that AS5Li30 possesses good cycling stability, with almost no degradation in water uptake. This makes it feasible to be used in applications triggered by solar energy and other low-grade energy resources.
5. Estimation results indicate that the applying of AS5Li30 in water harvester can produce a significant amount of fresh water and it displays wide environment adaptation.

## List symbols

A/ACFF: Activated carbon fiber felt
AWH: Atmospheric water harvesting
c: Fitting parameter of Eq. (3)
d: Fitting parameter of Eq. (3)
k: Fitting parameter of Eq. (2)
n: Fitting parameter of Eq. (2)
P: Pressure, Pa
R: Gas universal constant, J/(mol /K)
S: Silica sol
T: Temperature, K
W: Water uptake, g/g
${{x}}_{{0}}$: Fitting parameter of Eq. (2)

## Subscripts

ads: Adsorption
cond: Condensation
des: Desorption
LiCl: Lithium chloride
sat: Saturation
